# Clinical impact of PSMA PET/CT in primary prostate cancer compared to conventional nodal and distant staging: a retrospective single center study

**DOI:** 10.1186/s12885-020-07192-7

**Published:** 2020-08-05

**Authors:** Maarten L. Donswijk, Pim J. van Leeuwen, Erik Vegt, Zing Cheung, Stijn W. T. P. J. Heijmink, Henk G. van der Poel, Marcel P. M. Stokkel

**Affiliations:** 1 Department of Nuclear Medicine, The Netherlands Cancer Institute, Plesmanlaan 121, Amsterdam, the Netherlands 1066 CX; 2 Department of Urology, The Netherlands Cancer Institute, Amsterdam, the Netherlands; 3 Department of Radiology and Nuclear Medicine, Erasmus MC, Rotterdam, Netherlands; 4 Department of Radiology, The Netherlands Cancer Institute, Amsterdam, the Netherlands

**Keywords:** PSMA, Prostate, Staging, Management, Impact

## Abstract

**Background:**

To evaluate the impact of Gallium-68 [^68^Ga] labeled prostate specific membrane antigen (PSMA) positron emission tomography (PET)/X-ray computed tomography (CT) compared with conventional imaging on staging and clinical management of men evaluated for primary prostate cancer (PCa).

**Methods:**

Men with newly diagnosed biopsy-proven PCa who had been staged with a conventional staging protocol including bone scintigraphy (BS) and additionally underwent [^68^Ga]PSMA PET/CT, were evaluated retrospectively. Imaging findings from BS, magnetic resonance imaging (MRI) and/or CT were categorized regarding locoregional nodal (N) and distant metastasis (M) status as negative, positive or equivocal before and after addition of the information of PET/CT. Also, the imaging-based level of confidence (LoC) in correct assessment of N and M status was scored. Impact of PET/CT on clinical management was evaluated by the percentage of treatment category changes after PET/CT as determined in the multidisciplinary tumour board.

**Results:**

Sixty-four men with intermediate and high-risk PCa were evaluated. With additional information of PET/CT, N status was upstaged in 23%, and downstaged in 9%. M status was upstaged in 13%, and downstaged in 23%. A net increase in LoC of 20% was noted, mainly regarding M status.

Treatment category changed from palliative to curative in 9%, and from curative to palliative in 3%. An undecided treatment plan changed to curative in 14%, as well as to palliative in another 9%. In total, a 36% treatment category change was noted.

High negative predictive value of PET/CT for M status was indicated by 27 patients that underwent robot-assisted radical prostatectomy and reached postoperative biochemical disease-free status or had a likely other site of disease recurrence.

**Conclusions:**

PSMA PET/CT can cause considerable changes in N and M staging, as well as in management compared to conventional staging. Findings of this study support the replacement of BS and CT by PSMA PET/CT in staging primary PCa.

## Background

Management strategies for primary prostate cancer, whether with curative or palliative intent, have their own morbidities and costs. Recently estimated costs of prostate cancer therapies in Australia were US$ 15 K–35 K per patient and US$ 270.9 M in total, with an expected increase of 42% in 2025 [1]. Imaging has a pivotal role in staging of and selecting the appropriate management strategy in men with primary prostate cancer. Bone scintigraphy (BS) with Technetium-99 m [^99m^Tc] labeled bisphosphonates has been the most widely used method for detecting bone metastases of PCa, based on visualizing the increased osteoblastic activity of bone metastases [2]. Due to its moderate sensitivity and specificity, BS frequently results in equivocal findings regarding the presence of bone metastases [3–5]. Nonetheless, for primary staging of newly diagnosed PCa, BS is still recommended for distant staging, combined with cross-sectional abdominopelvic imaging for local and lymph node staging, in all high-risk patients [2].

After its clinical introduction in 2011, PET imaging with agents targeting the prostate-specific membrane antigen (PSMA), a transmembrane glycoprotein that is highly overexpressed on most PCa cells, has shown increasing adoption. Its value for staging recurrent PCa has already been well established [6].

More recently, PSMA PET/CT imaging was evaluated as a potential tool for staging primary prostate cancer in men prior to curative treatment [7]. Specificity rates of 84–100% were reported by earlier studies [8–11] and in a recent systematic review [12]. However, the sensitivity of PSMA PET/CT for the detection of lymph node metastases is moderate (33–91%), most likely due to the limitations in the spatial resolution to detect small (< 3 mm) lymph node tumour deposits in primary as well as recurrent prostate cancer [13]. Its performance with regard to distant metastases and impact on clinical management compared to conventional staging is less investigated*.*

In our institute, a Gallium-68 [^68^Ga] labeled PSMA PET/CT was added to the conventional staging procedure in men with intermediate and high-risk PCa (predominant Gleason pattern 4 or higher, and/or cT3 or higher, and/or PSA blood level ≥ 20) from June 2016 onwards. The aim of the present study is to evaluate the impact of additional PSMA PET/CT on staging and clinical management of men evaluated for primary PCa compared with a conventional staging protocol.

## Methods

### Ethics approval

This single-center was approved by the institutional review board of the Netherlands Cancer Institute and the need for written informed consent for usage of pseudonymized patient data was waived due to the retrospective nature of the study. Approval was registered under local number IRBd19063.

### Patients

For this study, all patients with newly diagnosed biopsy proven PCa who had been staged between June 2016 and February 2018 with both [^68^Ga]PSMA PET/CT and BS were identified and selected from an institutional database, containing staging and treatment information. According to institutional protocol diagnostic procedures were performed before start of any treatment although androgen deprivation therapy (ADT) at time of imaging was allowed. Maximum time between conventional imaging and PSMA PET/CT was 90 days, which has been suggested as an acceptable interval for comparing staging modalities in PCa [4].

### Conventional staging

Conventional staging included a clinical T stage assessment with digital rectal examination and PSA blood level measurement as well as conventional imaging including MRI, CT and BS. The MRI of the pelvis (multiparametric prostate protocol at a field strength of 1.5 T or 3 T, without an endorectal coil) was evaluated by experienced radiologists according to PI-RADS v2. Conventional size criteria thresholds were used for lymph node evaluation [14]. BS consisted of planar images of the entire skeleton from anterior and posterior 3–4 h after intravenous injection of approximately 555 MBq [^99m^Tc] labeled bisphosphonates, with additional detail views and / or SPECT/CT imaging of a body part if regarded necessary. All BS images were evaluated by experienced nuclear medicine physicians.

CT images were assessed for nodal and distant metastases. Generally, pelvic lymph nodes > 8 mm in maximum short axis diameter were regarded as positive. Either a contrast-enhanced abdominal CT (ceCT) was performed, or the lowdose CT (ldCT) as part of the PET imaging was used if no ceCT was performed. The ldCT was interpreted by two independent, experienced readers with clinical information but blinded from PET and other imaging results.

MRI, CT and BS findings were categorized according to the original clinical reports for regional nodal (N) as well as distant metastases (M) staging: negative, positive or equivocal. For ldCT, the readers reached a consensus on N and M stage using the same categories. A composed conventional N and M stage was determined grouping the results of the MRI, CT and / or BS. Positive status on one of the conventional imaging modalities was regarded dominant over equivocal and negative results from the other modalities. Equivocal status was regarded dominant over negative results. The rationale is that modalities are complemental and scan ranges may not overlap, therefore a result that upstages the patient has to be disproven by additional imaging or procedures, and / or to be judged true or false when determining appropriate therapy.

### PSMA PET/CT staging

Glu-NH-CO-NH-Lys-(Ahx)-[^68^Ga]-HBED-CC ([^68^Ga]PSMA-11) was used as tracer and produced on-site compliant to Good Manufacturing Practices regulations using a fully automated system (Scintomics GmbH, Germany). The tracer was administered to the patients as an intravenous bolus injection (100 MBq fixed dose). After an incubation period of 45 min, PET imaging was performed from proximal femora to skull base on a Philips Gemini TF-II PET/CT scanner (3 min / bed for pelvis/abdomen and 2 min / bed position for the remainder), combined with a dose-modulated low dose CT (40 mAs, 2 mm reconstruction).

All PET/CT images were interpreted by nuclear medicine physicians with experience in prostate cancer PET imaging and reporting. Level of tracer uptake, location and morphological appearance of lymph nodes were considered to assess N status [7, 15, 16]. PET/CT imaging findings were categorized according to the original reports for regional nodal (N) as well as distant metastases (M) staging: negative, positive or equivocal.

### Treatment policies

Cases were discussed as part of standard clinical care in the tumour board (consisting of urologists, medical oncologists, radiation oncologists, radiologists and nuclear medicine physicians) with all diagnostic information available including PSMA PET/CT. A formal TNM staging and a preferred treatment were recorded in patients’ chart. For patients with localized or regional lymph node metastases preferred treatment options with curative intent consisted of surgery or radiation (external or brachytherapy) whether or not combined with androgen deprivation. After therapy with curative intent, biochemical disease status was assessed with serum PSA measurements during follow up. Patients with high suspicion for distant metastases were considered for palliative treatment (ADT +/− chemotherapy).

For purpose of the study, cases were discussed again in a smaller expert group (MD, PvL, HvdP) without information from PSMA PET/CT and a fictional TNM staging and preferred treatment was recorded in the study database.

### Impact of PSMA PET/CT on staging

Per patient, the tumour N and M stage based on the composed conventional staging was compared to the tumour stage based on the additional information of the PSMA PET/CT. Differences in observed staging frequencies were tested for independence using a Chi-square test (IBM SPSS Statistics v25; Armonk, NY, USA). For both N and M staging, upstaging was defined as a change from negative to equivocal or positive, or from equivocal to positive. Downstaging was defined as a change from positive to equivocal or negative, or from equivocal to negative.

Furthermore, the level of confidence (LoC) in the correct assessment of the tumour stage based on the imaging findings was defined. This was done according to above mentioned N and M staging three-category systematics. For both N and M staging, increase in LoC was defined as a change from equivocal to positive or negative. Decrease in LoC was defined as a change from positive or negative to equivocal.

### Impact of PSMA PET/CT on clinical management

Based on the recorded tumour stage with and without information from PSMA PET/CT, patients were divided into one of three categories of intended treatment: ‘curative treatment’, ‘palliative treatment’, or ‘undecided’. ‘Undecided’ was assigned in case of equivocal M stage based on the conventional staging. The impact of PSMA PET/CT on clinical management was defined as a change of treatment category after additional PSMA PET/CT.

## Results

### Patients

**Fig. 1:**
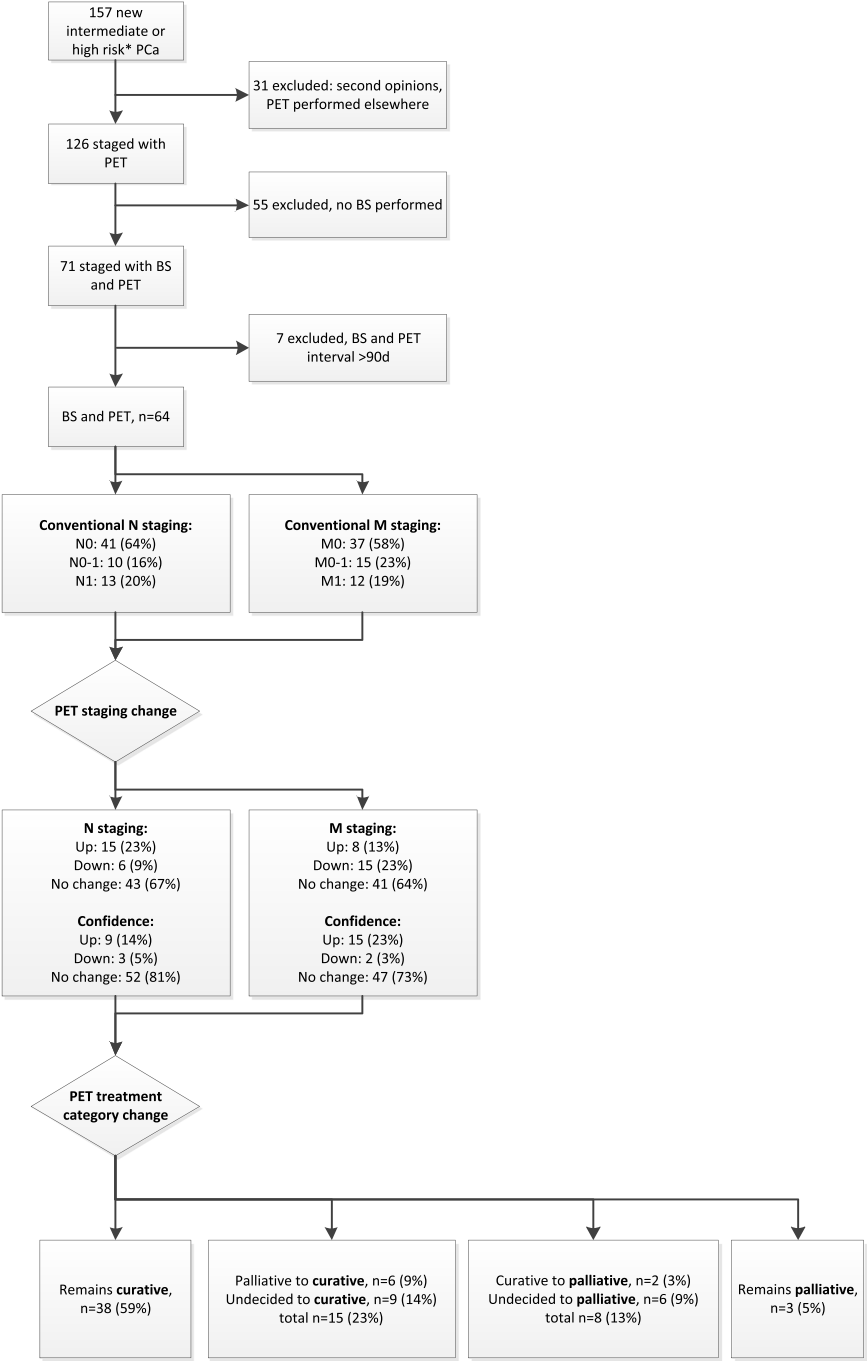
Flowchart of staging and treatment intent changes after PSMA PET/CT compared to conventional staging. * predominant Gleason pattern 4 or higher, and/or cT3 or higher, and/or PSA blood level ≥ 20

Sixty-four men with a BS and a [^68^Ga]PSMA PET/CT meeting the inclusion criteria were identified from the database (Fig. 1). Eight (13%) had intermediate-risk PCa and 56 (87%) had high-risk PCa. Four patients used the antiandrogenic oral drug bicalutamide and one patient used a gonadotrophin-releasing hormone analogue at time of PSMA PET/CT. Baseline patient characteristics are presented in Table 1. Performed imaging as part of the conventional staging protocol and median time between conventional imaging and PSMA PET/CT are presented in Table 2.

**Table 1:** Baseline characteristics

Total number of patients	64
Age in years, median (range)	69 (49–83)
PSA ng/ml, median (range)	17 (2,3–281)
**Clinical tumour stage**	
cT1c	6 (9%)
cT2a	8 (13%)
cT2b	7 (11%)
cT2c	17 (27%)
cT3a	8 (13%)
cT3b	11 (17%)
cT4a	4 (6%)
**Gleason score**	
6	3 (5%)
7a^a^	9 (14%)
7b^b^	8 (13%)
8	29 (45%)
≥ 9	15 (23%)
**Risk stratification**	
Intermediate risk	8 (13%)
High-risk	56 (87%)
**ADT use at time of PSMA PET/CT**	
Yes	5 (8%)
No	59 (92%)

^a^Predominant Gleason score 3

^b^Predominant Gleason score 4

**Table 2:** Imaging characteristics

Total number of patients	64
**Imaging performed**	
PSMA PET/CT	64 (100%)
Bone scintigraphy	
planar	64 (100%)
SPECT/CT	30 (47%)
MRI prostate^a^	59 (92%)
CT abdomen	
low dose	54 (84%)
contrast enhanced^b^	10 (16%)
Conventional imaging and PET/CT interval, median (range)	49 days (−12 to 87)

^a^in 5 patients no MRI available due to evident distant metastases on PSMA PET/CT [3], due to severe claustrophobia [1] and performed elsewhere [1]

^b^diagnostic abdominal CT with intravenous iodine-containing contrast in portal venous phase

### Impact on staging

Significant differences in N and M staging frequencies were found for CT and MRI, and for CT respectively compared to PSMA PET (Table 3). The linear weighted Kappa for the ldCT readers’ agreement was 0.5 for N stage and 0.58 for M stage. Based on conventional staging, 41 patients (64%) were staged as negative, ten (16%) as positive, and thirteen (20%) as equivocal for nodal metastases. With additional information of the PSMA PET/CT, 15 (23%) were upstaged, six (9%) were downstaged, and 43 (67%) remained unchanged.

**Table 3:** Comparison of conventional and PSMA PET/CT staging. *n* = 64 patients

**A. Staging frequencies**					
**N stage**		CT	MRI	Conventional^a^	PSMA PET/CT
negative		44 (69%)	43 (67%)	41 (64%)	38 (59%)
equivocal		9 (14%)	8 (13%)	10 (16%)	4 (6%)
positive		11 (17%)	8 (13%)	13 (20%)	22 (34%)
not available		–	5 (8%)^b^		
**M stage**	Bone scintigraphy	CT	MRI	Conventional^a^	PSMA PET/CT
negative	44 (69%)	54 (84%)	3 (5%)	37 (58%)	48 (75%)
equivocal^a^	9 (14%)	6 (9%)	5 (8%)	15 (23%)	3 (5%)
	• *M1b: 9 (14%)*	• *M1a: 2 (3%)*	• *M1b: 9 (14%)*		• *M1b: 3 (5%)*
		• *M1b: 4 (6%)*			
positive	11 (17%)	4 (6%)	0	12 (19%)	13 (20%)
					• *M1a: 2 (3%)*
					• *M1b: 9 (14%)*
					• *M1c: 2 (3%)*
not reported			51 (80%)		
not available^b^			5 (8%)		
**B. Contingency tables for observed staging frequencies**					
*N stage*					
	negative	equivocal	positive	Totals	
**Conventional**^a^	41	10	13	64	
**PSMA PET/CT**	38	4	22	64	
Totals	79	14	35	128	
*X*^2^ (2, *N* = 64) = 5.0; *p* = .0821					
	negative	equivocal	positive	Totals	
**CT**	44	9	11	64	
**PSMA PET/CT**	38	4	22	64	
Totals	82	13	33	128	
*X*^2^ (2, *N* = 64) = 6.0; *p* = .049076*					
	negative	equivocal	positive	Totals	
**MRI**	43	8	8	59	
**PSMA PET/CT**	38	4	22	64	
Totals	81	12	30	123	
*X*^2^ (2, N = 64) = 8.0; *p* = .018451*					
*M stage*					
	negative	equivocal	positive	Totals	
**Conventional**^a^	37	15	12	64	
**PSMA PET/CT**	48	3	13	64	
Totals	85	18	25	128	
*X*^2^ (2, *N* = 64) = 9.5; *p* = .008811*					
	negative	equivocal	positive	Totals	
**Bone scintigraphy**	44	9	11	64	
**PSMA PET/CT**	48	3	13	64	
Totals	92	12	24	128	
*X*^2^ (2, *N* = 64) = 3.3; *p* = .188193					
	negative	equivocal	positive	Totals	
**CT**	54	6	4	64	
**PSMA PET/CT**	48	3	13	64	
Totals	102	9	17	128	
*X*^2^ (2, *N* = 64) = 6.1; *p* = .046943*					

^a^composed conventional stage grouping results of bone scintigraphy, CT and MRI

^b^in 5 patients no MRI available due to evident distant metastases on PSMA PET/CT [3], due to severe claustrophobia [1] and performed elsewhere [1]

*denotes significance at alpha. 05 (Chi-square test of independence)

Based on conventional staging, 37 patients (58%) were staged as negative, 12 (19%) as positive, and 15 (23%) as equivocal for distant metastases. With additional information of the PSMA PET/CT eight (13%) were upstaged, 15 (23%) were downstaged, and 41 (64%) remained unchanged. A net increase in LoC of 20% was noted, mainly regarding M status. Further frequencies and changes in staging and LoC are presented in Tables 3 and 4.

**Table 4:** Impact of PSMA PET/CT staging and level of confidence on a per-patient level. *n* = 64 patients

**N stage**	N Status	LoC^a^
up	15 (23%)	9 (14%)
down	6 (9%)	3 (5%)
no change	43 (67%)	52 (81%)
**M stage**	M Status	LoC^a^
up	8 (13%)	15 (23%)
down	15 (23%)	2 (3%)
no change	41 (64%)	47 (73%)

^a^LoC: imaging-based level of confidence in correct assessment of N and M status

Twenty-seven patients underwent RARP. Eighteen of these reached biochemical disease-free status (defined as serum PSA < 0.01 after RARP without hormonal treatment), confirming absence of distant metastases as established with PET/CT staging. Notably, four of these patients were staged positive and two equivocal with conventional staging.

Nine patients did not reach postoperative biochemical disease-free status. Eight of these were categorized as M-negative, one as equivocal on PSMA PET. The most likely source of disease in these patients were remaining nodal metastases, because the majority of patients (8/9) had histologically proven nodal positive status after pelvic lymph node dissection (PLND) and follow-up PSMA PET/CT in all patients within a year after surgery (median 7 months, range 3–11) showed nodal metastases as the only site of recurrence in five patients, no localization in three patients, and multiple localizations including lymph nodes in one patient.

### Impact on clinical management

With additional information of the PSMA PET/CT, in 15 patients (23%) the treatment category changed from palliative (*n* = 6) or undecided (*n* = 9) to curative. In eight patients (13%) the treatment category changed from curative (*n* = 2) or undecided (*n* = 6) to palliative. In 41 patients (64%) the treatment category based on conventional staging did not change.

**Fig. 2:**
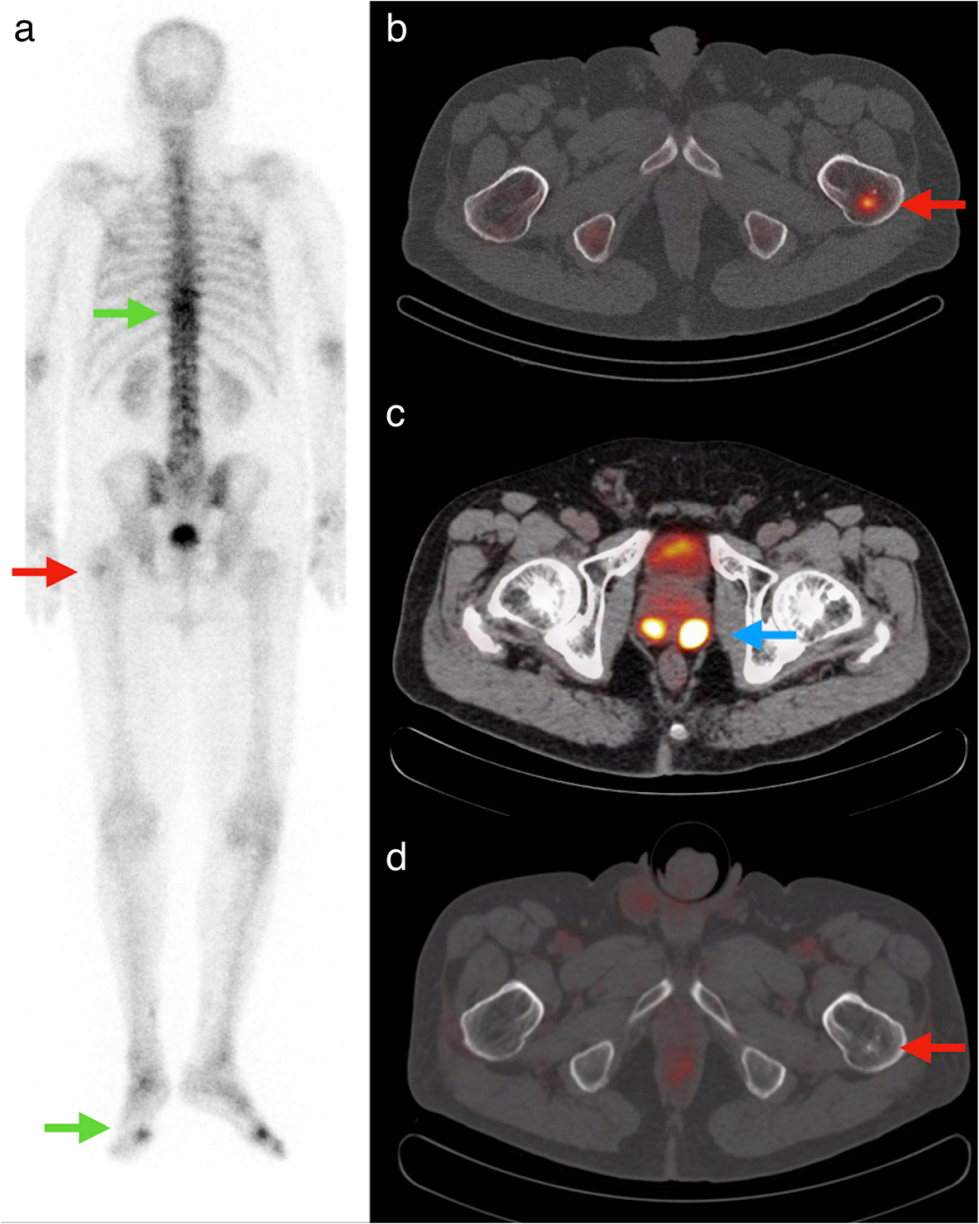
Example of staging change of PSMA PET/CT compared with conventional imaging. A man presented with a clinical T2c Gleason 7b (predominant Gleason score 4) PCa with an iPSA of 16. Planar BS (**a**) showed a faint spot in the left proximal femur (red arrow) which was confirmed on SPECT/CT (**b**) as a sclerotic lesion with osteoblastic activity (red arrow). The lesion was interpreted as suspicious for bone metastasis. Other areas with increased osteoblastic activity (green arrows) were interpreted as degenerative. A [^68^Ga]PSMA PET/CT (**c**) 36 days later shows a PSMA positive bilateral PCa (blue arrow), but no PSMA expression in the sclerotic lesion in the left proximal femur (**d**, red arrow). Based on PSMA PET/CT the lesion in the left proximal femur was regarded as not suspicious for bone metastasis; M stage changed from positive to negative and treatment intent changed from palliative to curative

The impact of PSMA PET/CT on treatment intent is presented in Table 5 and Fig. 1, and illustrated in Fig. 2.

**Table 5:** Treatment plan based on conventional vs PSMA PET/CT staging procedure. *n* = 64 patients

	Conventional staging	PSMA PET/CT staging	Impact of PSMA PET/CT	
Curative	40 (63%)	53 (83%)	Remains curative	38 (59%)
Undecided	15 (23%)	0	Palliative to curative	6 (9%)
Palliative	9 (14%)	11 (17%)	Undecided to curative	9 (14%)
			Undecided to palliative	6 (9%)
			Curative to palliative	2 (3%)
			Remains palliative	3 (5%)

## Discussion

In this study, the clinical impact of additional diagnostic information from PSMA PET/CT was compared with conventional staging in patients with intermediate and high-risk prostate cancer. A considerable impact on both staging and clinical management was found. Compared to conventional primary staging of PCa, PSMA PET/CT changed N stage in 33% and M stage in 36%, resulted in a net increase in the LoC of 9% for N status and of 20% for M status, and led to a treatment category change in 36%.

A growing number of studies compared PSMA PET/CT with conventional primary staging for primary PCa. Earlier studies found superior detection rates of PSMA PET/CT for bone metastases compared to BS in primary PCa patients [3, 4, 17]. Furthermore, PSMA PET/CT induced management changes were reported by Roach et al. in 21% of a larger cohort of primary PCa patients [18].

The recently published prospective multicentre proPSMA trial confirms the superior accuracy of PSMA PET/CT to conventional imaging (CT and bone scintigraphy) in assessing pelvic nodal and distant metastatic disease (92% vs 65%) in high-risk primary PCa [19]. Furthermore, equivocal findings were less (7% vs 23%) and PSMA PET/CT conferred more management changes (28% vs 15%). Also, when used as second-line imaging which is comparable to our study setting, PSMA PET/CT resulted in significant upstaging of nodal status compared to conventional imaging (23,3% vs. 8,8% positive) and management changed occurred in 27%.

The findings of our study are in line with abovementioned studies. In 33% of patients N stage was changed with information from PSMA PET/CT, mostly resulting in upstaging (23%). Although correctness of these findings could not be formally assessed in our study, the high reported specificity of PSMA PET/CT for nodal metastases in primary staging of PCa suggests that upstaging would be correct in most cases, warranting considerable changes in patient management, such as increasing the extent of the PLND or the radiotherapy field [8–11, 20, 21].

PSMA PET/CT-induced treatment category changes in our study ranged from a conservative 13% (curative to palliative, or vice versa) to an optimistic 36% (any treatment category change). This is in line with abovementioned studies as well. Prostate cancer management is likely to be more and more patient-tailored and adjusted to staging information of imaging, such as tumour delineation in radiotherapy planning which may be signficantly influenced by PSMA PET/CT [21]. Findings that may not result in a considerable management change now, may do so in the future. The LoC in determining the correct N stage was considerably higher with PSMA PET/CT compared to conventional staging, though equivocal results remained in up to 6%. This is comparable with abovementioned studies as well. Further development of structured reporting criteria for negative and positive nodes may help to reduce the number of equivocal results and increase the LoC [15].

The major limitation of our study is the retrospective nature. Impact of PSMA PET/CT may be overestimated because patients may have been more likely to receive a PSMA PET/CT because of inconclusive findings on conventional imaging. Patients with overt metastases on BS probably are underrepresented because PSMA PET/CT may have been omitted due to lack of clinical consequences, and this may have overestimated staging and management impact of PSMA PET/CT as well. However, this reflects clinical practice as BS is cheaper and more readily available and may serve as an adequate first line M staging tool in patients with highly elevated PSA levels. Furthermore, a true gold standard (histopathology) lacks in most cases, especially for distant metastases. The final diagnosis is substantially influenced by the findings of the PSMA PET/CT itself, leading to incorporation bias and overestimating the accuracy of PSMA PET/CT. The management changes described here however represent clinical practice. Although predefined rules were followed, the setting of the smaller unblinded expert group may have influenced the results of the fictional restaging and subsequent preferred treatment. Finally, the proportion of contrast-enhanced CTs in this study was low. Although literature showed poor sensitivity for CT in determining N stage and no established role in determining M stage in primary PCa, the comparison of PSMA PET/CT with conventional staging in our study may have been limited [22].

Use of ADT at time of PSMA PET imaging is allowed according to EANM procedure guideline on 68Ga-PSMA PET/CT imaging [23], however tracer uptake and therefore PET staging results may be influenced by ADT [24]. As only five of 64 patients were undergoing hormonal therapy at time of PSMA PET, we do not think this has impacted our results.

The purpose of this study was not to assess the comparative accuracy of the conventional staging and the staging with PSMA PET/CT. Since histopathological confirmation of N status in the non-surgical treatment group lacked and confirmation of M status clinically was not feasible or did not have consequences, a true gold standard was not available. However, a high negative predictive value of PSMA PET/CT for M status is indicated by the 27 patients treated with RARP who reached biochemical disease-free status or were likely to have alternative sites of residual disease. Notably four of these patients were staged positive and two of them equivocal with conventional staging method. Thus, in addition to reported higher sensitivity of PSMA PET/CT for bone metastases compared to BS in primary staging of PCa [4], our data indicate a superior specificity of PSMA PET/CT compared to conventional staging.

## Conclusions

Compared to conventional primary staging of PCa, PSMA PET/CT changes N stage in 33% and M stage in 36%, leading to treatment category change in 36%. BS was false-positive for distant metastases in 15–22% of cases. Findings of this study support the replacement of BS and CT by PSMA PET/CT in primary staging of PCa, but prospective analyses are needed to confirm a possible beneficial effect on survival outcome.

## Data Availability

The datasets generated and analysed during the current study are not publicly available as these contain individual person’s data but are available from the corresponding author on reasonable request, after pseudonymization of the data and legal agreement.

## Abbreviations

[^68^Ga]: Gallium-68
[^68^Ga]PSMA-11: Glu-NH-CO-NH-Lys-(Ahx)-[^68^Ga]-HBED-CC
[^99m^Tc]: Technetium-99 m
ADT: Androgen deprivation therapy
BS: Bone scintigraphy
ceCT: contrast enhanced X-ray computed tomography
CT: X-ray computed tomography
ISUP: International Society of Urologic Pathologists
ldCT: lowdose X-ray computed tomography
LoC: Level of confidence
N: Regional nodal
M: Distant metastasis; M1a non-regional lymph node, M1b skeletal and M1c visceral metastasis
MRI: Magnetic resonance imaging
PET: Positron emission tomography
PCa: Prostate cancer
PLND: Pelvic lymph node dissection
(i) PSA: (initial) prostate specific antigen
PSMA: Prostate specific membrane antigen
RARP: Robot assisted radical prostatectomy
